# The Relationship Between George Evelyn Hutchinson and Vladimir Ivanovic Vernadsky: Roots and Consequences of a Biogeochemical Approach

**DOI:** 10.1007/s10739-023-09717-9

**Published:** 2023-05-23

**Authors:** Pier Luigi Pireddu

**Affiliations:** Departamento de História e Filosofia das Ciências, Universidade de Lisboa – Faculdade de Ciências (FCUL) / Centro Interuniversitário de História das Ciências e da Tecnologia (CIUHCT), Campo Grande C4, Lisbon, Portugal

**Keywords:** History of ecology, History of limnology, Ecosystem, V. I. Vernadsky, G. E. Hutchinson, Biogeochemistry

## Abstract

Focusing on the relationship between two important scientists in the development of ecological thought during the first half of the twentieth century, this paper argues that Yale limnologist G. E. Hutchinson's adoption of the biogeochemical approach in the late 1930s builds on the 1920s work of the Russian scientist V. I. Vernadsky. An analysis of Hutchinson’s scientific publications shows that he first referred to Vernadsky in 1940, on two different occasions. This article analyzes the dynamics of Hutchinson’s formulation of the biogeochemical approach, providing historical context and linking its early application to the existing limnological tradition.

## Introduction

The question of *how* and *when* George E. Hutchinson came into contact with the work of Russian scientist Vladimir I. Vernadsky is the focus of this article—in particular, how the Russian's work influenced and, in some respects, contrasted with the research that Hutchinson conducted during his early time at Yale University as a limnologist. My intent here is not to establish a complete revisionist framework, but to address a narrower issue, limited to the late 1930s through the early 1940s, in order to situate the beginning of this relationship and to trace the first moment when Hutchinson systematized a biogeochemical approach by engaging with Vernadsky's work. The paper is organized into three sections. The first part offers a reconstruction of Hutchinson's early research period at Yale University, as well as biographical information useful for focusing the discussion. The second part deals with Hutchinson’s first encounter with the research work of Vernadsky, primarily through the works *La géochemie* and *La biosphere*. The third and final section goes into the relationship between the two scientists’ work, introducing Hutchinson’s definition of the biogeochemical approach (Hutchinson 1940; Hutchinson and Wollack 1940) and the context of his research at Linsley Pond.

According to Hutchinson and Wollack (1940), limnologists such as Birge and Juday in America and Thienemann in Europe first introduced a biogeochemical approach which, however, finds its more advanced form in Vernadsky's work. In commenting on *Bio-Ecology* Hutchinson (1940) stated that a general neglect of biogeochemistry was due to the fact that organisms and environment were typically not considered part of the same ecological unit. The biogeochemical approach opened the way for investigations that include the biota and physicochemical factors as part of the same system. Hutchinson’s contribution was to bring biogeochemical complexity within a limnological context, during a period straddling Tansley's (1935) proposal of the ecosystem concept and Lindeman's (1942) work—for McIntosh (1985, p. 125) a “watershed in ecology.” The essence of the ecosystem concept, the idea of a perspective that can consider the interactions between organisms and the non-living environment, can partly be traced to Vernadsky, together with a practical way of investigating ecological systems in terms of energy and material exchange.

However, the argument here will be limited to reconstructing the emergence of the biogeochemical approach in Hutchinson's research, and to providing historical-conceptual context to frame Vernadsky as one founder of the ecosystem concept as it matured. A biogeochemical approach would emerge in Hutchinson’s analysis of ecological succession in lake systems, with Linsley Pond the central study site.

## A Distinguished Limnologist at Yale University: The Early Career of George Evelyn Hutchinson

To introduce the argument, it is necessary to start with Hutchinson's biography. After fine-tuning some biographical details, I will briefly review some of the scientific work Hutchinson conducted during the years 1938–1941.

### Biographical Notes

Hutchinson was born in Cambridge, England in 1903 and graduated with a degree in Zoology from University of Cambridge in 1925. As his first research experience he spent a short period in Naples at the Anton Dohrn Zoological Station, working on the endocrine systems of octopus and squid. The following year he moved to South Africa to the University of Witwatersrand in Johannesburg, where he began to learn about limnology. It was during these years that he conducted his first research on lake systems, a passion that later made him one of the leading experts in this discipline. As a product of his experience in South Africa (Carruthers 2011), he published his first limnological works (Hutchinson 1928a, b; Hutchinson 1929, 1931, 1933; Hutchinson et al., 1932).

He did not spend much time in Johannesburg, as he learned from his friend and colleague Lancelot Hogben of the possibility of a Seesel Anonymous Fellowship at Yale University. As a result, Hutchinson arrived in New Haven, Connecticut in 1928 and spent his entire academic career at Yale.

It was not long after his arrival at Yale University, in the late 1930s, that Hutchinson learned of the research work of Russian scientist Vladimir I. Vernadsky through some relationships he formed at Yale. In particular, it was George Vernadsky and Alexander Petrunkevitch that acquainted Hutchinson with the Russian scientist's work. The former was Vladimir's son, who worked at Yale teaching Slavic languages, while the latter was a zoology student of V. Vernadsky himself and the son of one of his closest friends (Hutchinson 1991; Slack 2010). The three formed a bond that was more than professional. During the years 1944 and 1945, G. Vernadsky and Petrunkevitch, with the help of Hutchinson, published translations of two of the senior Vernadsky's most influential papers: “Problems in biochemistry, II: The fundamental matter-energy difference between the living and the inert natural bodies of the biosphere” (1944) and “The biosphere and the noosphere” (1945). This was a significant occasion, according to Hutchinson in his autobiography: “Vernadsky had a strong influence on some aspects of my research, and I did my best to help Petrunkevitch and George Vernadsky make their ideas about biosphere better known in English-speaking countries” (Hutchinson 1979, p. 233). It was the first time that the works of the Russian scientist were disseminated in the United States of America (Oldfield and Shaw 2013).

Vernadsky's research was important to Hutchinson's work in ecology, as I will show. In particular, the new biogeochemical science inaugurated by Vernadsky directly influenced some areas of his research, fostering the tendency to analyze certain ecological phenomena from a physicochemical point of view (Grinevald 1987, 1996; Taylor 1988; Hagen 1992; Cooper 2003).

Furthermore, Vernadsky himself was aware of the work of the American scientist. As pointed out by A. Lapo, in correspondence with B. L. Lichkov, Vernadsky remarked “My son's friend, Hutchinson, who has much to do with geochemistry and biogeochemistry and has several followers now, has initiated a new branch in this field” (Lapo 2001, p. 48). This new branch, as I will discuss below, found its origins in the early work at Linsley Pond.

### The Scientific Research and the Activity at Linsley Pond

George Hutchinson's biography is well documented (Slobodkin 1993; Slobodkin and Slack 1999; Slack 2010; Lovejoy 2011), and it is not necessary to go into further detail beyond his encounter at Yale with George Vernadsky. However, a detailed discussion of some of his research activity before the end of World War II is appropriate. Hutchinson first referred to Vernadsky's work on two separate occasions in 1940, in connection with his limnological analyses.

**Fig. 1:**
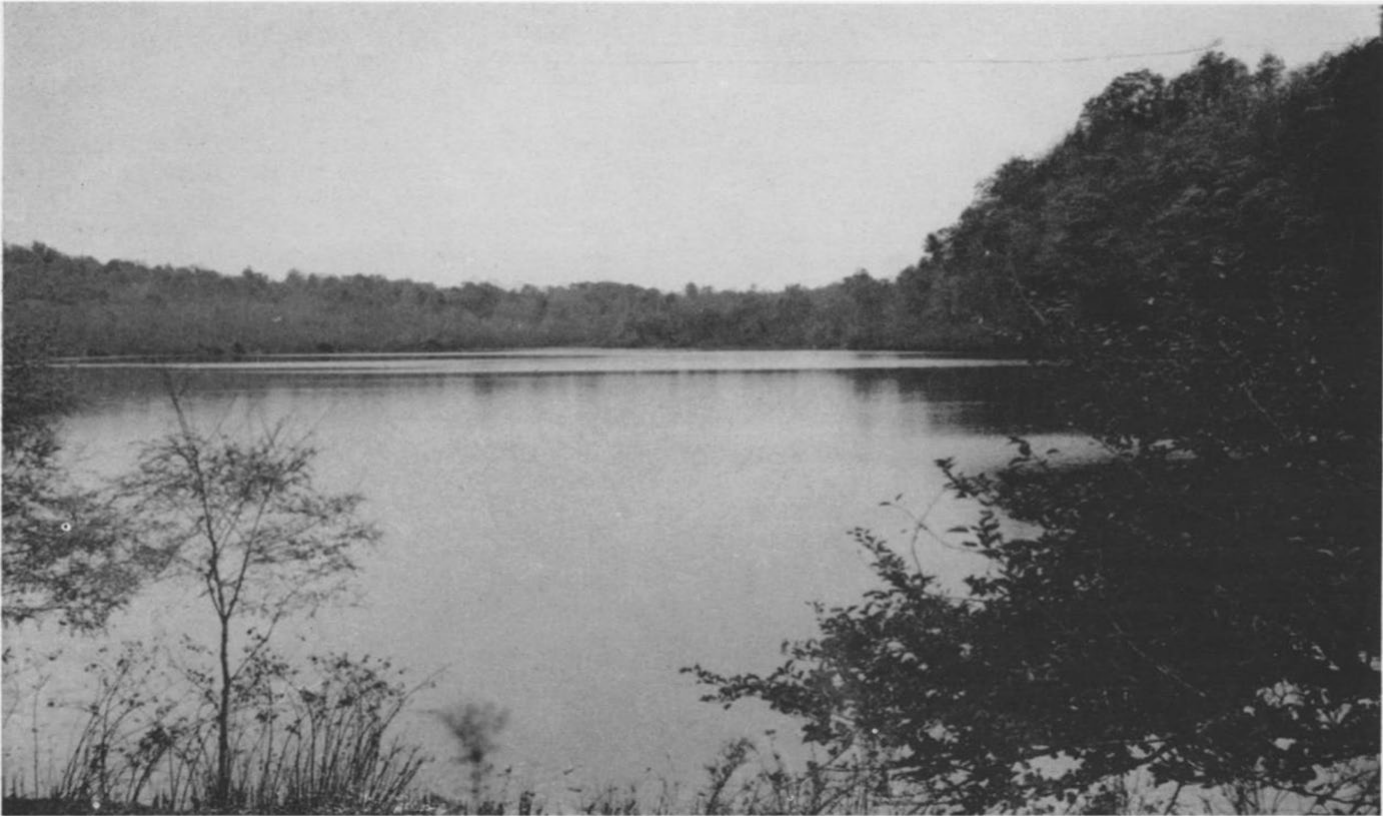
Linsley Pond, Connecticut, USA (from Hutchinson 1941, p. 24)

Between 1938 and 1941 five articles were published by Hutchinson, two of them written in collaboration with his students. During this brief period Hutchinson was most influenced by the research of A. Thienemann, one of the most distinguished European limnologists.^1^ Further insights came to Hutchinson from two pioneers of limnology, E. A. Birge and C. Juday, who worked in the United States.^2^ Most of Hutchinson’s research was conducted at Linsley Pond, a lake in North Branford, Connecticut (Fig. 1).

In 1938 Hutchinson published two articles, “Chemical stratification and lake morphology,” and “On the relation between the oxygen deficit and the productivity and typology of lakes” (Hutchinson 1938a, b). The first of these discussed the “nature of the water movements in the hypolimnion of thermally stratified lakes” (Hutchinson 1938a, p. 63). It was a topic which had previously received considerable attention. In fact, Birge and Thienemann, along with A. Grote, had already investigated the “nature of such water-movements” (Hutchinson 1938a, b, p. 64). In the second article, Hutchinson provided an analysis of the distribution and deficit of oxygen in the epilimnion and hypolimnion, an index affecting biological phenomena in the related aqueous zone. In this study Hutchinson built on the mostly descriptive work previously carried out by Birge and Juday (1911).

Hutchinson’s article of 1939, “The oxidation–reduction potentials of lake waters and their ecological significance*,*” co-authored with E. S. Deevey and A. Wollack drew heavily on the research of Thienemann. Specifically, it consisted of an analysis of how the benthic Chironomid fauna varied in various lake systems, and how these species reacted in the presence of particular chemical conditions. Indeed, it was Thienemann (1913, 1920, 1922) who first drew attention to the *Chironomidae* as an ecological criterion in lake classification.

For present purposes, Hutchinson’s article of 1940, “Studies of Connecticut lake sediments II: Chemical analyses of a core from Linsley Pond, North Branford,” written in collaboration with A. Wollack, is the most relevant.^3^ It was there that Hutchinson mentioned Vernadsky’s name for the first time in his scientific writings. I will discuss the context of the article and further analyze its contents below. However, it is important to note here that the name of the Russian scientist does not appear among the bibliographical references. Rather, Hutchinson referred to Vernadsky while introducing the biogeochemical approach, distinguishing it from a bio-sociological approach. According to McIntosh (1985), this was the first time that this methodological dichotomy was identified in the history of ecology. This dichotomy emerged after the development of the ecosystem concept, with its agenda to describe the reciprocal interactions between physical and chemical agents and organisms. Thus, Hutchinson and Wollack described the bio-sociological approach as based on species and their relationships, whereas the biogeochemical approach was based on the study of material and energy transfers within a volume of space, and highlighted the importance of the differing results that followed from the two methodologies. The authors endorsed the biogeochemical approach.

Hutchinson returned to Vernadsky's work in 1943.^4^ In Hutchinson’s 1941 “Mechanisms of intermediary metabolism in stratified lakes” the name of the Russian scientist is not mentioned; instead the analysis was based on studies done by Birge and Juday (1911, 1929, 1934). Comparing the results obtained by them in Lake Mendota, Wisconsin (Egerton 2016), with those he obtained in Linsley Pond, Hutchinson's article offers an analysis of alkalinity and temperature, and discusses the movement or cycle of nutrients within the lake system of Linsley Pond (see 3.2).

During this first phase of his limnological scientific research, Hutchinson referred explicitly to Vernadsky's works only once. I will soon explore the context in which he did this, and the debate into which the biogeochemical approach was introduced.

## Biogeochemical Science: Vladimir I. Vernadsky

First it is necessary to provide some background on Vernadsky’s career as well as essential aspects of his scholarly output. Here I begin by introducing the figure of Vernadsky, pointing out some key biographical events. Then I highlight some major points about his two books *La biosphere* and *La géochemie*. These two works represent the connection with Hutchinson, as it was there that Vernadsky systematized the guidelines of the new biogeochemical science.

### Science Without Boundaries: Vladimir I. Vernadsky

Vernadsky was born in St. Petersburg, Russia on March 12, 1863. A graduate of the University of St. Petersburg in 1885, Vernadsky began to devote himself to mineralogy. Nevertheless, his scientific profile can be described as exceptionally broad. Several biographies on Vernadsky's life have already been written (Lapo 2001; Edmunds and Bogush 2012; Tagliagambe 2017), covering different aspects of his activities as a researcher.

His formative period at the University of St. Petersburg, where he first matriculated in 1881, was particularly influential and fruitful, as he was surrounded by outstanding scientists who also played a role in his professional training, among them Dmitrij Ivanovic Mendeleev and Vasilij Vasil'evic Dokucaev. As Tagliagambe (2017) explains, while the former had an influence on Vernadsky as a point of reference for the general theme of his work, the latter was his major mentor. Dokucaev was the one who directed Vernadsky to the study of geospheres and introduced him to geochemistry, a field of investigation that kept Vernadsky occupied for his entire career.^5^

Early in his career, from 1885 to 1888, Vernadsky worked as a minerologist, first as Curator of Mineralogy at St. Petersburg University later leaving Russia to work at prestigious research centers in Europe, mainly in Italy, Germany, France and Great Britain—working, among many others, with such personalities as crystallographer Arcangelo Scacchi (1810–1893), mineralogist Paul Heinrich von Groth (1843–1927), and chemist Henri Louis Le Châtelier (1850–1936). He returned to Russia in 1890, teaching courses in mineralogy and crystallography at Moscow University. In the early twentieth century, Vernadsky organized a Commission on the Study of Natural Resources of Russia for the Russian Academy of Sciences, and served as the first president of the Ukrainian Academy of Sciences from 1918 to 1919. In 1922 he founded, in Moscow, the Radium Institute and 6 years later the Biochemical Laboratory (BIOGEL), currently known as the Vernadsky Institute of Geochemistry and Analytical Chemistry of the Academy of Sciences.

The 1920s are the most important period for our present purposes. In December 1921, Vernadsky was invited to teach a course in geochemistry at the Sorbonne in Paris. He travelled with his wife and daughter to the French capital, where he stayed until 1925 with brief interruptions for travel and study in other countries, notably Czechoslovakia and England. The period at the Sorbonne yielded important results. Already in the preceding years Vernadsky had paid special attention to the question of organic matter and its relations to the lithosphere, atmosphere and hydrosphere (Edmunds and Bogush 2012), as well as on the role of organisms in the cycles of chemical elements. Influenced also by the academic environment he encountered in Paris (where he came into contact with internationally notable figures such as Edward Leroy and Pierre Teilhard de Chardin), these insights took shape and led to a systematized and coherent scientific account in two publications: *La géochemie* (1924)*,* his collected lectures at the Sorbonne from 1923 and 1924, and *La biosphère* (1929), in which he presented a broader, more systematic development of the same ideas (Grinevald 1987, 1996).

The question of living matter and its role within global balances, at the chemical level, and its relationship to the non-living domain is the central topic of *La géochemie* and *La biosphère*. In a sense, life on Earth was not a random phenomenon, but was embedded within a systemic context. Vernadsky was thus the first scientist who outlined the characteristics of a research approach that relate environment (e.g., lakes, soils, seas) to the domain of life, where living organisms are not mere spectators but act directly influencing planetary processes and their evolution (Grinevald 1993; Grinevald and Polunin 1988; Tagliagambe 1994, 2017). In this sense, his was an approach that broke down barriers between domains, and pushed toward disciplinary interaction, in search of a more complete picture of natural phenomena. The organism–environment relationship thus found a broader framework, where it was not just a function of the adaptation of the former to the latter, but a more encompassing one, interpreting the environment as something continuous with the organisms themselves. Besides geochemistry, the new biogeochemical science introduced by Vernadsky addressed the study of the chemical composition of living matter and its role in geological processes globally, attending to the migration of chemical elements through different spheres, including the living domain.

Biogeochemistry became, and remains, an important field in ecological research (Odum 1953; Likens 1995), and has been recently described as the first multi-disciplinary science (Bianchi et al. 2021). Vernadsky is now recognized as its founder (Grinevald 1996; Kautzleben and Müller 2014; Tagliagambe 2017; Egerton 2019).

### On the Development of the New Biogeochemical Science and the Concept of Biosphere

Among Hutchinson's sources were *La biosphere* and Vernadsky’s 1939 article “On some fundamental problems of biogeochemistry.” In the latter, Vernadsky proposed a definition of the term biogeochemistry, which Hutchinson (1943) cited to describe the discipline:

Biogeochemistry, which is a part of geochemistry and has peculiar methods and peculiar problems of its own, may be finally reduced to a precise quantitative mathematical expression of the living nature in its indissoluble connection with the external medium, in which the living nature exists. A living organism thus acquires an aspect different from the one it has in biology; it is expressed in numbers of atomic or weight composition, in physical, quantitatively expressed, manifestations of the space it occupies, in numeral energetical expressions of the work it does in the space of life upon our planet. Life in the biogeochemical aspect is the living matter of the biosphere, that is, the total of all the living organisms present in the biosphere at a given moment. Thus, the living organism itself, expressed in numbers, is a new independent expression of the same phenomenon, which the biologist views in a vivid physiological and morphological expression of the innumerable forms of life. Between these precise and scientific expressions of life relations might be and should be sought for. (Vernadsky 1939, p. 1939)

Thus, biogeochemistry turned out to be a specific field of geochemistry.

In *La géochemie* (1924), Vernadsky sketched a detailed account of the envisioned discipline, placing it among those (such as physics of the atom, radiology and radiochemistry), which dealt with atoms. It is important to point out that Vernadsky highlighted the link between organic matter and inert matter, emphasizing how these two domains intrinsically connected (Vernadsky 1924, p. 42). Moreover, Vernadsky insisted on the need to investigate organisms and their role in material exchange with the environment. Moreover, with Vernadsky:

The main processes of living organisms that are clearly related to their surrounding environment - breathing and nutrition - are studied without their repercussions in the surrounding environment from which organisms retrieve and return chemical elements. (Vernadsky 1924, p. 45, trans. author)^6^

The concept of *cycle* is thus emphasized, in terms of the incessant exchange at the atomic level among living organisms, mainly through the processes of respiration and nutrition. This was a cycle that involved both living matter and the inorganic domain, for example in the Earth’s carbon cycle,^7^or in the case of iodine and bromine. In the chapter discussing the geochemical history of iodine and bromine, *Histoire del'iode et le brome*, Vernadsky offered the following reconstruction, where the notion of the cycle was made explicit:

At the Earth's surface, iodine and bromine are dispersed, their free atoms or ions are sought by living organisms and are concentrated in compounds formed by them which contain, for example, those of some marine sponges 8.5 percent of the iodine and probably more. It seems that a part of these atoms is retained by superficial chemical reactions and gives vadose minerals. It is very likely, however, that these chemical reactions can only occur in more or less continuous relation to living matter, since they are observed under conditions favourable to the accumulation of organic matter, the product of living matter. Over time, the iodine and bromine products of the organisms, as well as the vadose minerals are destroyed, the iodine and bromine return to the state of atoms and ions to start again the same cycle. (Vernadsky 1924, pp 41–42, trans. author)^8^

The concept of the cycle is also echoed in Hutchinson's early activity at Linsley Pond (see 3.2). Vernadsky envisioned material cycles driven by life on the grandest scale. This was a view that emerged not only in *La biosphere*, as discussed below, but also a few years earlier in *La géochemie*. With Vernadsky:

The small, invisible living beings produce the most considerable effects. This current of biological research was soon completely dried up, but the set of ideas that composed it is now revived in geochemistry. The action of living beings in the history of the chemical elements of the earth's crust is produced in large part by their nutrition and respiration. In geochemistry the organisms manifest themselves and can be studied only in the overall effect produced by these physiological processes, the whole of which forms a planetary phenomenon. (Vernadsky 1924, pp. 47–48, trans. author)^9^

In this way, recognizing the planetary scope of living matter and its influence in geochemical processes, Vernadsky already in *La géochemie* laid the groundwork for the later volume *La biosphère* (Grinevald 1996; Levit 2001; Rispoli 2015; Egerton 2019). In it, as noted, the indivisible relationship between inorganic matter and living matter was recognized. Life and inert matter represent a unitary system (Rispoli 2015), a step towards *bio*geochemistry (Tagliagambe 2017), where the two domains (living and non-living) were now juxtaposed. In other words, for Vernadsky, biogeochemistry was the discipline that confered a common perspective and established a link between living matter and inert matter.

Anticipating, in some features, the notion of global ecosystem (Huggett 1999), Vernadsky proposed a perspective where the biosphere was included within a cosmic picture, intimately related to solar action. Specifically, the biosphere was the *envelope* that covers the Earth,^10^ its outer area that separated it from celestial space, within which life develops. It was the purpose of biogeochemical science to focus on the essential interaction between living and nonliving within that envelope. In the 1939 article “On some fundamental problems of biogeochemistry,” this assumption emerged clearly:

Life is continuously and immutably connected with the biosphere. It is inseparable from the latter materially and energetically. The living organisms are connected with the biosphere through their nutrition, breathing, reproduction, metabolism. This connection may be precisely and fully expressed quantitatively by the migration of atoms from the biosphere to the living organism and back again — the biogenic migration of atoms. (Vernadsky 1939, p. 43)

The function of life within the mechanism of the biosphere was therefore indispensable to the stability of the chemical balance of the planet (Oldfield and Shaw 2013). With biogeochemistry, Vernadsky sought to broaden the spectrum of relationships, from organic interactions to those with the nonliving environment.

In the appendix to *La biosphere* entitled “The evolution of species and living matter” (*L'évolution des espèces et la matière vivante*) Vernadsky introduced some essential principles of his biogeochemical science. The French text reports the following:

Life is an integral part of the biosphere mechanism. This is clearly evident from the study of the geochemical history of chemical elements, since biogeochemical processes, which are so important, always require the intervention of life.

These biogeochemical manifestations of life constitute a set of vital processes absolutely distinct at first sight from those studied by biology. (Vernadsky 1929, p. 203)^11^

Such a contextualization and integration of the organic world with the principles of physics and chemistry led to a new interpretation of living organisms. Through a biogeochemical approach, the organism could be defined as an aggregation of atoms and considered in quantitative terms through concepts such as the average chemical composition of a living being.^12^ Thus, an incessant cycling of chemical elements occured within the biosphere, which the organism assimilated from, and released into, the environment, resulting in a complex process of transformation and not just assimilation by organic matter. The French edition concluded as follows:

The study of biogeochemical phenomena, pushed as far as possible, allows us to explore precisely the field of the dependent manifestations of life and of the physical structure of the universe, as well as the domain of future scientific theories.

This explains the deep philosophical interest that the biogeochemical problems present nowadays. (Vernadsky 1929, p. 230, trans. author)^13^

It is this conceptual structure that was introduced in the USA in the late 1930s by Hutchinson: a renewed approach toward the study of the domain of life, in its inevitable connections with the physicochemical factors of the environment.

## Between George E. Hutchinson and Vladimir I. Vernadsky

This concluding section is structured into two main parts. In the first one, the first definition of the biogeochemical approach given by Hutchinson in his 1940 article with Wollack is discussed, along with his second use of the concept in a review of Clements and Shelford's book *Bio-Ecology* (1939). In short, Hutchinson found in Vernadsky's biogeochemical approach a unifying and systematic framework that could accommodate the limnological tradition familiar to him, particularly the works of Birge, Juday, and Thienemann. In the second part of this section I offer a detailed analysis of some ways in which the Russian scientist influenced Hutchinson’s approach to limnological research at Linsley Pond.

### On the Definition of a Biogeochemical Approach

On two different occasions in 1940, Hutchinson referred to Vernadsky's biogeochemical research, in one instance, emphasizing the paternity of the term biogeochemistry (Hutchinson and Wollack 1940), and in the other referring simply to the biogeochemical approach in a critical review (Hutchinson 1940).

In the 1940 article co-authored with Wollack, the biogeochemical approach was used to analyze small-scale systems within limnological research (Certomà 2008). Specifically, Hutchinson and Wollack (1940) highlighted several issues regarding the development of organic sediments in Linsley Pond and the function organic matter performs in the process of eutrophication of lacustrine systems (see 3.2). Introducing biogeochemistry in this context meant highlighting the inevitable relationships or co-relationships between biota and physicochemical factors, then expanding on those dynamics. As noted above, Hutchinson and Wollack drew a sharp distinction between the biogeochemical approach and the bio-sociological approach.^14^ The latter only allowed the investigation of “the history of individual units and their interactions” (Hutchinson and Wollack 1940, p. 510), and thus focused narrowly on species and organic relationships:

This has been the underlying thought behind the bio-sociological part of ecology, in spite of the protests of synecologists that the community is an organism and is to be studied as a whole. That such a particulate bias has been introduced unconsciously is probably due to the obviousness of the individual units and the training of biologists in the taxonomic recognition of species. The association is therefore defined quantitatively in terms of relative numbers of different species. (Hutchinson and Wollack 1940, p. 510)^15^

The biogeochemical approach was presented as a remedy for this traditional bias. The two methodologies were not described as complementary in scientific practice and—as explained by Taylor (1988)—only later did Hutchinson (1948) attempt to integrate them. The following is the excerpt of considerable relevance:

This second method consists in isolating a suitable volume of space, either naturally (e.g., the whole earth, a lake) or arbitrarily (e.g., a cubic meter of seawater or living matter over an acre of forest) defined, and then studying the transfer of matter and energy in a given time across the boundaries of this volume. This method is the starting point of thermodynamics and has been immensely powerful in the physical sciences. It is implicit in much of physiology and biochemistry, as in the study of tissue metabolism by means of respiratory quotients. In the development of such an approach an estimate of the total amount of living matter included in the volume under consideration is of greater importance than an estimate of its taxonomic diversity. Ideally, of course, both methods should be used together; in practice, however, this is often impossible. As a result of the neglect of what may be called, using Vernadsky's term, the biogeochemical approach, some branches of ecology, particularly the study of succession, seem to have suffered. (Hutchinson and Wollack 1940, pp. 510–511)

This Vernadskian viewpoint would directly influence his limnological work, encouraging him to investigate ecological phenomena from the perspective of physics and chemistry, thus connecting biological and abiotic factors in terms of material and energy exchange.

This new effort to focus on biomass and not on taxa, and on the interactions between living organisms and the abiotic environment is evident, for example, in Hutchinson’s studies of the phosphorus cycle in lakes systems (see 3.2). Hutchinson and Bowen (1947, 1950) tried to understand how the activity of the organic component affected the compensation of chemical cycles and compared changes in biological productivity with changes in nutrient availability (Taylor 1988). In the biogeochemical approach as developed by Hutchinson, physical and biological processes were strongly connected within a single scientific perspective. This perspective includes the sum of physical, chemical, and biological factors in a dynamic rather than static view, and it was from Vernadksy that Hutchinson introduced it to Western ecological science.^16^

Nonetheless, something like a biogeochemical perspective was not entirely alien to limnologists prior to Hutchinson. In fact, it is in their research that it is possible to find the roots of this approach (Gorham 1991; Bianchi 2020), which Vernadsky’s thinking helped Hutchinson develop into a more comprehensive analysis. Hutchinson suggested as much in a note:

This mode of approach has long been recognized in limnology and as far as the difficulties inherent in moving water masses permit, in oceanography. A less rigorous, if simpler, statement of the two methods may be found in the introduction to Birge and Juday’s (1911) monograph on the dissolved gases of the Wisconsin lakes (see also Thienemann 1939). (Hutchinson and Wollack 1940, p. 510)^17^

It was the same perspective integrating the inevitable connection between organisms and the environment that emerges in Hutchinson's commentary on Clements and Shelford’s *Bio-Ecology* (1939).^18^ Hutchinson’s most serious criticism of the work was that it treated organisms and the abiotic environment as distinct and separate matters:

The gravest defect of the book, in the reviewer's opinion, lies in its total neglect of certain very important approaches to the subject, in which its technical language is of no assistance. If, as is insisted, the community is an organism, it should be possible to study the metabolism of that organism. The neglect of this aspect of ecology, and of the fact that the living matter of the whole earth may be considered as a unit of higher order than the biome, leads the authors to make the following extraordinary statement, “from the very nature of the medium, the reactions of plants upon the air are usually less definite and controlling than upon the soil.” Photosynthesis is discussed briefly on the next page, but no idea of the fact, apparent to Joseph Priestley on the conclusion of his experiments, is given that here we are in the presence of the greatest controlling reaction of them all. This neglect of the biogeochemical approach is due in part to the authors' insistence that the community and the environment must be separated and should not be considered as forming part of the same ecological unit. (Hutchinson 1940, p. 268)

However, the idea of a connection between organisms and environment as inseparable units, or as a system in the sense of physics, had already been conceptually developed by Tansley (1935), although in a different context.^19^ Hutchinson played an important role in introducing the ecosystem concept into American ecology and, through his Vernadskian perspective, making matter and energy cycles central to ecosystem studies (Grinevald and Polinin 1988; Taylor 1988; Grinevald 1996). Effectively, Hutchinson was able to provide the Clementsian community structure with a solid physicochemical ground, studying its metabolism in terms of energy interactions and nutrient cycles, cycles that can be interpreted as “the province of Vernadsky's new science of biogeochemistry” (Cooper 2003, p. 63). Thus, through Hutchinson, Vernadsky’s ideas were incorporated into modern ecosystem theory (De Laplante 2005; Lefkaditou 2010; Oldfield and Shaw 2013).^20^

Hutchinson's source was the original French edition of Vernadsky's *La biosphère* published in Paris in 1929 (see 2.2). In the work, the Russian scientist introduced the volume as follows:

The purpose of this book is to draw the attention of naturalists, geologists, and especially biologists to the importance of the quantitative study of life in its indissoluble relationship with the chemical phenomena of the planet. (Vernadsky 1929, p. IX, trans. author)^21^

It is in the final appendix that Vernadsky most fully expounded his biogeochemical perspective. The following statement is noteworthy:

In biogeochemical processes, matter and energy are in the foreground instead of the form inherent to the species. From this point of view, the species can be considered as a matter analogous to the other matters of the earth's crust, such as water, minerals and rocks, which, along with organisms, are the object of biogeochemical processes. From this point of view, the biologist's species can be considered as a homogeneous living matter, characterized by mass, elementary chemical composition and geochemical energy. (Vernadsky 1929, p. 206, trans. author)^22^

Given this introduction to biogeochemistry, Vernadsky tightened the focus to the scientific approach toward living organisms:

In this field we consider, from the point of view of physical chemistry, organisms as autonomous fields where determined atoms are gathered in a determined quantity.

This quantity constitutes precisely the distinctive property of each organism, of each species. It indicates the number of atoms that the organism of a given species can retain outside the scope of the biosphere, due to its own force and therefore removed from the surrounding environment. The volume of the organism and the number of atoms it contains, expressed numerically, give the most abstract and simultaneously the most real formula of the species insofar as it is reflected in the geological processes of the planet. This formula is obtained by measuring the dimensions of the organism, its weight, and its chemical composition. This number of atoms and the volume of the organism thus determined are undoubtedly characteristics of the species. (Vernadsky 1929, p. 207, trans. author)^23^

Vernadsky thus urged a distinct approach to organisms: not as biological entities and investigated in their biotic interactions and in their continuous evolutionary process, but as a complex of atoms interacting with the surrounding environment. Hutchinson would embrace and develop the biogeochemical approach as an extension of the limnological tradition, with Thienemann, Birge, and Juday as his main points of reference.

### Linsley Pond: Between Limnology and Biogeochemistry

Hutchinson's limnological studies at Linsley Pond offered a multidisciplinary, complex and systemic perspective on the lacustrine environment (Fogg 1992). In the various studies Hutchinson addressed a number of technical issues. In the 1940 paper, a central ecological problem—that of succession—was discussed in a limnological perspective, incorporating biogeochemical complexity.

It was Hutchinson’s student E. S. Deevey who from 1935 dealt with the question of sediments in the bottom of Linsley Pond (Deevey 1939, 1940, 1941). The research continued for a decade during which Deevey was accompanied by Hutchinson and other students and colleagues, producing an extensive scientific literature. The nature of these sediments, first analysed by Deevey (1939) and then by Hutchinson and Wollack (1940), were mainly of an organic nature and demonstrated certain changes throughout the history of the lake system. Indeed, this organic material was particularly significant, and an analysis of its distribution shed light on the various changing occurrences in the lake’s development process, as reported by Hutchinson and Wollack (1940). This distribution clarified how there was a rapid transition from an oligotrophic to a eutrophic condition, followed by a long trophic equilibrium period manifested by producing a sediment of relatively constant composition. Through analogy, this mode of development of the biocenosis or community of the lacustrine system could be carefully compared to that of a single organism, as Hutchinson and Wollack indicated in their article:

It is impossible to avoid qualitatively comparison of this mode of development of the rate of organic production of sediment, and so within certain limits of development of the mass of organisms present, to the growth curves of individual organisms and homogeneous populations. (Hutchinson and Wollack 1940, p. 508)

Hutchinson—as historian Joel Hagen (1992) shows—never worked within the Clementsian paradigm. Nevertheless, he recognized the heuristic potential of this association at the conceptual level and on this occasion with Wollack discussed the issue.

Comparing the growth curve of a biocenosis with that of a single organism (or a genetically homogeneous population) raised some difficulties. First, the nature of the constituents in one case had a certain continuity with predecessors, presenting a tendency toward a geometrically based growth (through a balance between opposing, multiplying and limiting forces, depicted in the so-called sigmoid curve of growth). In a biocenosis, in contrast, there was no necessary continuity within populations and there may have been no genetic continuity between organisms; geometric growth was less evident in this context. Therefore, it was the action taken by predecessors during the course of ecological succession, suggested Hutchinson and Wollack (1940), that must be considered in its full hereditary scope, producing a similar effect in terms of growth and changing the environmental context to support more organic matter. This was a colonization process, so it may be called, and was also evident in the dynamics of soil formation and nitrogen fixation. Thus, it was not the actions of individuals alone along with their relations that were crucial in the succession process. It was the different phenomena taking place within a defined environmental context—for example, a lake system—that had a function in the different stages of development. And it was this interplay of factors that Hutchinson and Wollack wanted to highlight: a multivariable system that had been underestimated because of what was called a *bio-sociological bias*, not accounting for a possible and inevitable relationship between organisms and the environment in which they lived and behaved. In short, the problem of ecological succession, in a limnological context, opened discussion in more than strictly biological terms.^24^

Hutchinson and Wollack concluded, through estimation of the amount of organic matter in lake bottom sediments (much of it of planktonic origin and deposited by other organisms inhabiting the lake) and study of its distribution along its profile, that the lake underwent a period of rapid acceleration in productivity and an immediately following period of trophic equilibrium. This conclusion was deduced from a preliminary consideration that took a new perspective toward the study of living organisms. No longer defined in terms of biological diversity, but in terms of the mass—and here Vernadsky's influence emerged clearly (in relation to the numerical constants, mass, chemical composition and geochemical energy, for the study of ecosystems)—present in a given volume, so that nutrient exchanges across inorganic (sediment and water) and organic components of the system could be delineated. (Here Vernadsky’s influence emerged, again, in terms of the capacity of the organism to retain atoms by removing them from the environment.) Therefore, two conclusions followed from the early Hutchinsonian biogeochemical approach: (1) the sudden growth that invested the development of the biocenosis in Linsley Pond; (2) the subsequent period of trophic equilibrium. These events were the essential features of the Linsley Pond succession process.

Within a lake system, what is called *trophic equilibrium* is derivable from an analysis of the biogeochemistry of the lake and is comparable, in a sense, to the concept of *climax formation* for plant ecologists. In this ecological context, biogeochemical dynamics such as nutrient exchanges and cycling between water and sediment were indispensable for understanding the successional process. The entry within the lake system of elements such as phosphorus and nitrogen directly affected ecosystem productivity, leading to internal imbalances and altering a potential trophic equilibrium condition. Therefore, according to Hutchinson and Wollack, trophic equilibrium could be determined by the balance between nutrient inputs and outputs, with relative productivity limited mainly by phosphorus and nitrogen. With regard to phosphorus, Hutchinson gave the following argument:

In the case of phosphorus, a very large part of the total mass entering planktonic organisms at any time is derived from the mud, and is returned to the latter by sedimentation of organic debris. The small amount of phosphorus that is lost permanently to the sediment is replaced by a small excess of the phosphorus entering over that leaving the lake. (Hutchinson and Wollack 1940, p. 513)

The phosphorus scheme (already discussed by Vernadsky in *La biosphère*), in the context of the limnological analysis developed by Hutchinson and Wollack, fell within the biogeochemical approach. It was the study of the transfer of nutrients and materials within a well-defined volume of space, and the cycle accomplished by these elements through the different components of the system, integrating different areas of expertise. This was not just a biological investigation, but a spectrum of inquiry that involved chemical and physical complexity.^25^ The reduction of organisms to the chemical level allows for a definition of the migration of elements through the trophic levels of a lake system, establishing how nutrient excesses and deficiencies lead to consequences in the balance of an ecological system. Hutchinson and Wollack pointed out the issue, in concluding their analysis:

The final equilibrium would probably be determined in all cases by the small balance of incoming nutrients in the inlet over the outgoing nutrients in the outlet. Since phosphorus is essential to nitrogen-fixing bacteria and algae as to other organisms, and all the other biological elements are doubtless present in excess, it is probable that it is the phosphorus income that is the fundamental limiting factor. (Hutchinson and Wollack 1940, p. 516)

Therefore, it was an equilibrium condition established by the action of the limiting factors, which was definable from the cycles that nutrients make through ecological systems.^26^ Hutchinson and Wollack (1940) suggested, concluding the article, that benthic fauna had to be considered the most intuitive factor in the displacement of phosphorus in the aquatic environment, through their metabolic activity.

Thus biogeochemistry opened new perspectives for investigation into ecological succession, finding in limnology direct application on the (debated) question of trophic balance. If, according to Vernadsky, biogeochemistry studied the role that living organisms play in circulating chemical elements, and extended this perspective into the relationship between organic matter and the entire biosphere, then Hutchinson reshaped the approach by confining it to a limited and defined volume, Linsley Pond, seeking to draw conclusions about the impact that the presence or absence of certain elements (especially phosphorus and nitrogen) may have on the overall system.

## Conclusion

The relationship between Hutchinson and Vernadsky carries with it several implications for the history of ecology. Starting from my reconstruction of some of Hutchinson’s research, I have highlighted in the course of this paper the moment when the Yale limnologist introduced, for the first time in his scientific writings, the basic concepts of Vernadsky’s biogeochemistry.

The year 1940 proved to be particularly significant: it was, in fact, on the occasion of the article written in collaboration with Wollack that Hutchinson mentioned Vernadsky's name for the first time. He did so by contrasting a biogeochemical approach to a bio-sociological approach, drawing a sharp methodological distinction with regard to ecological investigations. And it was in this circumstance that the relationship between the two scientists’ work took shape. This occurred in a particular historical moment, between Tansley's (1935) proposal of the ecosystem concept and Lindeman's (1942) first formulation in reference to a lake system. Hutchinson, in fact, intervened in the debate on Clements’s concept of superorganism—commenting that same year on the work of Clements and Shelford’s *Bio-Ecology* (1939)—and positioning himself in the same conceptual lineage that Tansley had extended by coining the term ecosystem. Hutchinson’s intervention emphasized the need for a perspective, a methodological approach, which considered organisms and the abiotic environment as a single unit. For Hutchinson, Vernadsky's biogeochemistry opened the possibility of analyzing from the point of view of physics and chemistry the exchanges of energy and material within a defined space. This was an approach already familiar to limnologists prior to Hutchinson. Therefore Hutchinson extended a fruitful approach in the limnological tradition, while drawing on the ideas and assumptions developed by Vernadsky to highlight the link between the domain of life and physical and chemical factors. In this way, the ecosystem concept was further defined. Biogeochemistry offered new insights into the question of ecological succession in limnology around 1940.

In conclusion, I would like to highlight a historical conjunction. Hutchinson's introduction of Vernadsky's biogeochemical perspective into limnology drew attention away from particular species and onto the elements within them. This offered new perspectives and a renewed interpretation of the dynamics of living organisms in the field of ecosystem ecology. Furthermore, it is a conjunction that helps us understand how Vernadsky's works were inserted for the first time in the American ecological debate, with Hutchinson as the main channel of transmission.

## Footnotes

1 Thienemann's role in promoting the activity of limnological scientific research in Europe was certainly relevant (Rodhe 1969; Egerton 2008; Jax 2020). In 1909 Thienemann began his research activity, describing the characteristics of the benthic fauna and publishing the first articles after having studied lake systems in various European regions. Laying the foundations for a work of classification (Moss et al. 1994), he defined two types of lakes: the first, characterized “by *Tanytarsus* in the sub-alpine regions of Germany” (Moss et al. 1994, p. 2), and the second, which are characterized by “*Chironomus* in the lowlands uncovered by the postglacial retreat of the Baltic Sea” (Moss et al. 1994, p. 2). This topic was further investigated by Hutchinson et al. (1939).

2 Birge and Juday were two pioneers in limnology between the nineteenth and twentieth centuries. The first collaborative paper, entitled “A summer resting stage in the development of *Cyclops biscuspidatus*,” was published in 1908. In the early years of the twentieth century, Birge and Juday began a thorough and systematic study of Wisconsin lake systems, believing that a collaborative, multidisciplinary approach was the key to a comprehensive understanding of lake dynamics and processes (Egerton 2015).

3 That article is part of a collection of scientific publications conducted at the southern Connecticut region, called the *Limnological Studies in Connecticut*. The first article in this collection is by Gordon A. Riley (1939), which continued with the participation of several authors—such as E. S. Deevey—for more than a decade.

4 In Hutchinson and Wollack (1940), Vernadsky was  informally referred to throughout the article; Hutchinson first explicitly and formally cited Vernadsky in 1943, including *La biosphere* among the bibliographical references in a paper in the *Quarterly Review of Biology*, entitled “The biogeochemistry of aluminium and of certain related elements*.*” It is considered by E. Gorham as “one of the most thorough explorations of plant uptake in biogeochemical terms” (Gorham 1991, p. 208). There Hutchinson acknowledged Vernadsky as originator of the term “biogeochemistry,” referring also to Victor M. Goldschmidt:: “To the present writer, the most fruitful approach to the fundamental problems of ecology lies in a synthesis of the results of Vernadsky, Goldschmidt, and certain other investigators entirely outside the traditional field of the biological sciences” (Hutchinson 1943, p. 2).

5 Dokucaev might be regarded as one of the pioneers of a systems approach, which the disciple Vernadsky inherited. Dokucaev was a geologist and geographer, normally known as a founder of soil science (Tagliagambe 1994), and he developed the first classification of soils. And it was his soil science—or pedology—showing a systems approach, that inspired Vernadsky. In Dokucaev's scientific work soil is seen as a living entity undergoing continuous transformation through a combined set of climatic and biological factors (Moon 2005). Thus, within the same scientific framework, climatic, plant, animal, and temporal elements intersect in order to provide a comprehensive overview. Biogeochemistry itself, which is strongly interdisciplinary, traces this same conceptual trajectory.

6 Original French: Les grandes processus des organismes vivants qui sont en relation évidente avec le milieu ambiant—leur respiration et leur nutrition—sont étudiés sans leur répercussion dans le milieu environnant d'où les organismes par leur intermédiaire tirent les éléments chimiques et les y rendent.

7 One example, broadly explained by Vernadsky within the geochemical history of carbon is what is called—in Vernadsky's terminology—the *cycle vital* (Vernadsky 1924, p. 312). Therein, a dynamic equilibrium between living matter and—with Vernadsky—carbon dioxide (CO_2_), which stand between them in a relationship whereby as the amount of carbon dioxide increases, the amount of living matter increases in turn. This is a process that is not entirely reversible, where only some of the absorbed carbon dioxide is returned to the environment, while most of it is retained by living matter, and when it is released it is absorbed in turn by other organisms (which vary their uptake capacity). In contrast, carbon that is not retained within the *cycle vital* is released in the form of “biogenic sub-carbonate minerals” (Vernadsky 1924, p. 312). Through this example, it is intended to reinforce the idea of the environment–organism relationship made possible through a process of reducing the living domain in atomic terms. It is no longer referred to as a *living organism*, but as *organic matter*.

8 Original French: A la surface terrestre, l'iode et le brome sont dispersés, leurs atomes ou ions libres sont recherchés par les organismes vivants et se concentrent dans des composés formés par eux qui contiennent par exemple ceux de quelques éponges marines 8,5 p. 100 de l'iode et probablement plus. Il semble qu'une partie de ces atomes est retenue par des réactions chimiques superficielles et donne des minéraux vadoses. Il est très probable cependant que ces réactions chimiques ne peuvent se produire qu'en relation plus ou moins directe avec la matière vivante, car elles s'observent dans des conditions propices à l'accumulation de la matière organique, produit de la matière vivante. Au cours du temps, les produits iodiques et bromiques des organismes, ainsi que les minéraux vadoses se détruisent, l'iode et le brome retournent à l'état d'atomes et ions pour recommencer de nouveau le même cycle.

9 Original French: Les tres vivants, petits, invisibles, produisent des effets les plus considérables. Ce courant des recherches biologiques fut bientôt tout à fait tari, mas l'ensemble des idées qui le composait revit maintenant dans la géochimie. Car l'action des êtres vivant dans l'historie des éléments chimiques de l'écorce terrestre est produite en plus grande partie par leur nutrition et leur respiration. En géochemie les organismes ne se manifestent et ne peuvent être étudiés que dans l'effet sommaire produit par ces phénomènes physiologiques, dont l'ensemble forme ainsi un phénomène planétaire.

10 Vernadsky returns to the concept of the *envelope* several times (1924, 1929, 1944, 1945), and later Hutchinson described the biosphere as the “envelope” of the Earth (Hutchinson 1970, p. 45).

11 Original French: La vie constitue une partie intégrante du mécanisme de la biosphére. C'est ce qui ressort nettement de l'étude de l'histoire géochimique des éléments chimiques, dus processus biogéochimiques, si importants, exigeant toujours l'intervention de la vie. Ces manifestations biogéochimiques de la vie constituent un ensemble de processus vitaux absolument distincts à première vue de ceux qu'étudie la biologie.

12 Seeking to broaden the scientific perspective through which the study of life is approached, Vernadsky elaborated as follows: “Thus the species is usually considered in biology from the geometrical point of view; the form, the morphological characters, occupy the first place there. In the biogeochemical phenomena, on the contrary, this one is reserved to the number and the species is considered from the arithmetical point of view” (Vernadsky 1929, p. 204, trans. author). [Ainsi l'espèce est habituellement considérée dans la biologie du point de vue géométrique; la forme, les caractères morphologiques, y occupent la première place. Dans les phénomènes biogéochimiques, au contraire, celle-ci est réservée au nombre et l'espèce est considérée du point de vue arithmétique]. For a biogeochemical perspective on life, otherwise, what must be focused on are numerical constants relating to average weight, average elemental chemical composition and geochemical energy—thus the relative ability of the organism to produce, according to Vernadsky (1929), atomic migrations to the external environment (Grinevald, 1993).

13 Original French: L'étude des phénomènes biogéochemiques, poussée le plus avant possible, nous fait pénétrer précisément dans le domaine des manifestations connexes de la vie et de la structure physique de l'univers, et, en même temps, dans celui des futures théories scientifiques. On s'explique le profond intérêt philosophique que présentent actuellement les problèmes biogéochemiques.

14 This distinction (out of the limnological context of Linsley Pond) is redeveloped in Hutchinson (1948) and rediscussed by Taylor (1988). In particular, with regard to a biogeochemical approach: “When a circular causal system involving a group of organisms is described in terms of the transfer of some substance through the system, without employing any purely biological enumeration, such as the size of a population, the mode of approach will be characterized as biogeochemical” (Hutchison 1948, p. 221). With regard to a bio-sociological (or now bio-demographic): When a circular causal system is described in terms of the variation in numbers of biological units or individuals, or, in other words, in terms of the variation in the sizes of populations, the mode of approach is characterized as biodemographic (Hutchinson 1948, p. 221).

15 In this case, in agreement with McIntosh, Hutchinson and Wollack “provided a suggestion of the organismic emphasis which appeared in ecosystem ecology” (McIntosh 1985, p. 198), by remarking how the growth curve of a lake system shows similarities to that of a single organism (see paragraph 3.2). Throughout his career, however, Hutchinson was critical of the organismic emphasis as developed by Clements, preferring an analytical-quantitative approach to communities over a classificatory one (Hagen 1993). One can assume that on a heuristic level such an analogy can be fruitful, in Hutchinson's view, but the analysis of community metabolism must be the focus of attention.

16 Oldfield and Shaw (2013) identify Hutchinson as one of the four most influential factors in the dissemination of Vernadsky's works in the West.

17 The reference to Birge and Juday is the work “The inland lakes in Wisconsin: the dissolved gases of the water and their biological significance” (Birge and Juday 1911). The work by Thienemann that he cited was *Grundzuge einen allgemeinen Oekologie* (Thienemann 1939).

18 The essence of Clements's (1905, 1916) theory consists in the concept of super-organism (Tobey 1981; McIntosh 1985, 1999; Worster 1994; Eliot 2007) as a term for plant communities, which—just like individual organisms—change over time through a system of progressive development. However, his concept of super-organism was revised in his elaborations and in other debates, further revisions. See van der Valk (2014) and Kirchhoff (2020).

19 Tansley describes his concept of an ecosystem as follows: But the more fundamental conception is, as it seems to me, the whole system (in the sense of physics), including not only the organism-complex, but also the whole complex of physical factors forming what we call the environment of the biome-the habitat factors in the widest sense. […]. It is the systems so formed which, from the point of view of the ecologist, are the basic units of nature on the face of the earth. Our natural human prejudices force us to consider the organisms (in the sense of the biologist) as the most important parts of these systems, but certainly the inorganic “factors” are also parts – there could be no systems without them, and there is constant interchange of the most various kinds within each system, not only between the organisms but between the organic and the inorganic. These ecosystems, as we may call them, are of the most various kinds and sizes. (Tansley 1935, p. 299). This topic has been widely studied (McIntosh 1980; Simberloff 1980; Hagen 1992; Golley 1993; Likens 1995; Looijen, 2000; O’Neill 2001; van der Valk 2014; Egerton 2017).

20 According to K. Jax, Vernadsky “had substantial influence on the development of an ecosystem theory related to material fluxes” (Jax 1998, p. 114); see also Taylor (1988) and DeLaplante (2006).

21 Original French: Le but de ce livre est d’attirer l’attention des naturalistes, des géologues, et sortout celle des biologists sur l’importance de l’étude quantitative de la vie dans ses rapports indissolubles avec les phénomenes chimiques de la planète.

22 Original French: Dans les processus biogéochimiques ce sont la matière et l'énergie qui sont au premier plan au lieu de la forme inhérente à l'espèce. L'espèce peut à ce point de vue être considérée comme une matière analogue aux autres matières de l'écorce terrestre, comme les eaux, les minéraux et les roches, qui, avec les organismes, sont l'objet des processus biogéochimiques. Vue sous cet angle, l'espèce du biologique peut être envisagée comme une matière vivante homogène, caractérisée par la masse, la composition chimique élémentaire et l'énergie géochimique.

23 Original French: Dans ce domaine nous considérons, du point de vue de la chimie physique, les organismes comme des champs autonomes où sont réunis des atomes déterminés en quantité déterminée. Cette quantité constitue précisément la propriété caractéristique de chaque organisme, de chaque espèce. Elle indique le nombre d'atomes que l'organisme d'une espèce donnée peut retenir en raison de la force qui lui est propre hors du champ de la biosphère et retirer ainsi du milieu ambiant. Le volume de l'organisme et le nombre d'atomes qu'il comporte, exprimés numériquement, donnent la formule la plus abstraite et en même temps la plus réelle de l'espèce dans la mesure où celle-ci se reflète dans les processus géologiques de la planète. On obtient cette formule en mesurant les dimensions de l'organisme, son poids, sa composition chimique. Ce nombre d'atomes et le volume de l'organisme ainsi déterminé sont indubitablement des caractères de l’espèce.

24 With respect to the axis between Hutchinson and Wollack (1940) and Vernadsky (1929), this new approach toward the study of the living domain is highlighted. Vernadsky distinguished a “biological approach,” which consisted of determining the characteristics of living organisms from a geometrical and morphological point of view, from a biogeochemical one. Hutchinson distinguished between a bio-sociological approach, which consisted of studying the history of organisms and their relationships, and a biogeochemical one. In both cases, the break (or broadening) of this interpretive framework finds greater continuity when biogeochemistry intervenes to expand the framework. On the one hand, Vernadsky insisted on numerical constants (such as total mass quantity, chemical composition, and geochemical energy), where organisms are agglomerations of atoms that are released and absorbed through the environment. The organism, in this way, was incorporated into the geological processes of the planet. On the other hand, Hutchinson proposeed that the study of the amount of living matter in a given environmental context (thus a lake) is a necessary condition for implementing a biogeochemical approach to the study of ecological systems.

25 Lindeman (1942) referred to the biogeochemical approach introduced by Hutchinson and Wollack (1940): From this viewpoint [the trophic-dynamic approach introduced by Lindeman in his work], which was closely related to Vernadsky's “biogeochemical” approach (cf. Hutchinson and Wollack, '40), a lake considered as a primary ecological unit in its own right, since all the minor “communities” mentioned above depend on other components of the lake food cycle […] for their very existence. Considering the trophic cycle further, the discrimination between living organisms as parts of the “biotic community” and dead organisms and inorganic nutrients as parts of the “environment” appears arbitrary and unnatural (Lindeman 1942, p. 399). Therefore, the idea of an inevitable and fundamental connection between organic and inorganic emerges. This underscores the influence Hutchinson had on Lindeman by introducing him to the works of Vernadsky (Cook 1977).

26 The limiting factor problem was also addressed in 1940 by Riley who, however, referred to Hutchinson and Wollack (1940) on the issue of nutrients (Riley 1940, p. 296).
